# Time-of-flight fluorescence depth mapping using a spatiotemporal deep learning model

**DOI:** 10.1117/1.JBO.31.6.066003

**Published:** 2026-06-18

**Authors:** Shiru Wang, Arthur Pétusseau, Claudio Bruschini, Edoardo Charbon, Petr Bruza

**Affiliations:** a Dartmouth College, Thayer School of Engineering, Hanover, New Hampshire United States; b Advanced Quantum Architecture Laboratory, EPFL, Neuchâtel, Switzerland

**Keywords:** time-of-flight, deep learning, fluorescence, topology, single-photon avalanche diode sensor

## Abstract

**Significance:**

Fluorescence-guided surgery (FGS) utilizes molecular contrast agents to highlight critical structures or pathological tissues in real time. The premise of FGS is to enable precise surgical decision-making through accurate visualization and quantitative assessment of fluorophore distribution. However, strong effects of diffusion and absorption of fluorescent light in tissue confound fluorescence images, preventing accurate quantitative assessment of the concentration and distribution of fluorescent markers. These optical artifacts may lead to misinterpretation of tissue boundaries and compromised surgical precision, thereby diminishing the capabilities of FGS. Resolving topological depth maps of fluorophore distribution at the millimeter scale is an important first step in performing quantitative sub-surface fluorescence imaging.

**Aim:**

In this study, we present a spatiotemporal deep learning architecture that utilizes picosecond single-photon avalanche diode (SPAD) sensor images to rapidly recover the depth topology of a fluorophore distribution embedded in diffuse media. The network is designed to work with wide-field, epi-illumination geometry and millimeter spatial resolution.

**Approach:**

A ConvLSTM-UNet deep learning network was developed for picosecond time-resolved image analysis. This network was trained on 5000 spatiotemporal maps simulated by the optical Monte Carlo method and convolved with the instrument response function (IRF) of the imaging system. The experimental setup utilized a SwissSPAD2 sensor synchronized with a 635 nm picosecond laser diode. Using only 10 selected temporal gates as input, the network could recover depth maps. Reconstruction accuracy was evaluated using mean error metrics across various depths and background concentrations of a fluorophore with a simulated decay time of 100 ps.

**Results:**

A total of 75 different test fluorescence video data were evaluated. This set encompassed 15 unique inclusion shapes at five different depths. The network successfully reconstructed fluorescence topography up to 15 mm with a mean absolute error of less than 0.6 mm and mean depth variances below 0.5 mm. The inference time was ∼30  ms.

**Conclusions:**

Integrating temporal and spatial deep learning networks enabled depth mapping from time-resolved fluorescence data. Utilizing real IRF proved the applicability of SPAD sensors for sub-surface fluorescence mapping.

## 1 Introduction

Fluorescence-guided surgery (FGS) is a well-established method used in various surgical and oncology procedures.^1^^–^^4^ It employs a fluorescent contrast agent along with an optical visualization system to highlight cells and tissues that are undetectable by the naked eye of the surgeon. FGS is currently established for tissue perfusion and vital structure imaging, as well as for the removal of residual tumor in certain types of cancer.^5^^–^^8^ This technique enhances the completeness of tumor resection^2^ by reducing positive margins^2^^,^^9^ and minimizing unnecessary excision of healthy tissue, thereby improving patient outcomes and reducing negative side effects.^1^ One of the major limitations of FGS is the ill-posed problem of fluorescence light transport, which is significantly affected by the scattering and absorption properties of different tissues. Furthermore, the fluorescence signals from inclusions at certain depths that contribute to the resulting image also strongly depend on the optical properties of tissue. To achieve a similar level of standardization as in other established diagnostic imaging systems such as computed tomography (CT), FGS requires a robust implementation of real-time tissue optical property correction that so far has not been solved. Until then, the resulting fluorescence images presented to the surgeon will rarely represent the true topological maps of fluorophore concentration, ultimately preventing FGS from achieving its main premise, i.e., to improve the objectivity of surgical resection.^10^^–^^12^

Current clinical fluorescence cameras cannot provide sufficient information for fluorophore topological mapping. First-order corrections of fluorescence brightness to minimize absorption effects were achieved using a spectral approach^13^^,^^14^ or by employing scanned or wide-field hyperspectral imaging.^15^^–^^17^ The majority of subsurface fluorescence topology mapping studies have utilized diffuse optical tomography (DOT) and experimentally similar fluorescence molecular tomography (FMT) approaches. However, these approaches require spatial arrangement of sources and detectors that are not readily translatable to a typical wide-field epi-illumination geometry in clinical FGS. CT priors have also been used to improve fluorophore localization, but similar to DOT/FMT, this method is applicable mainly to pre-clinical research. Spatial frequency domain imaging has demonstrated promising results in recovering optical properties of tissue and decoding fluorophore depth,^18^^–^^20^ yet the need for pattern scanning often leads to motion artifacts, which limits its clinical translatability.

Time-domain fluorescence imaging represents a promising approach to solving the diffuse optical inverse problem. In the 1990s, authors developed light transport theory based on analytical solutions of the radiative transport equation,^21^^,^^22^ which was implemented later to reconstruct topology and/or lifetimes using various iterative numerical optimization approaches.^23^ The early depth reconstruction methods localized depth by triangulating arrival-time features in wide-field temporal point spread functions^24^ or by fitting instrument response function (IRF)-convolved diffusion kernels,^25^ followed by various iterative approaches typically involving the Jacobian matrix inversion.^26^ The iterative solutions, however, suffer from the tradeoffs between forward model accuracy and computational burden, which limits their accuracy in complex scenarios.^27^

Recent developments of deep learning network methods have demonstrated superior performance and very short reconstruction time in depth-resolving fluorescence imaging. Zhang et al.^28^ developed a convolutional neural network (CNN)-based fluorescence molecular tomography approach, which has shown faster and more accurate reconstructions without the reliance on solving an explicit forward model. Smith et al^29^ demonstrated that CNNs extract accurate fluorescence lifetimes from picosecond-resolved image stacks. However, the presented design is limited to per-pixel fluorescence decay recovery and does not account for the spatial distribution or neighboring pixel signal mixing.^29^^,^^30^ This approach, however, may suffer from complications near fluorophore concentration boundaries, where diffused light may be misidentified as false positive without prior knowledge of topology. Recently, Nizam et al.^31^^,^^32^ presented a promising approach that combined time-resolved wide-field imaging with k-space pattern illumination and a CNN model for 3D fluorescence lifetime reconstruction.

**Fig. 1:**
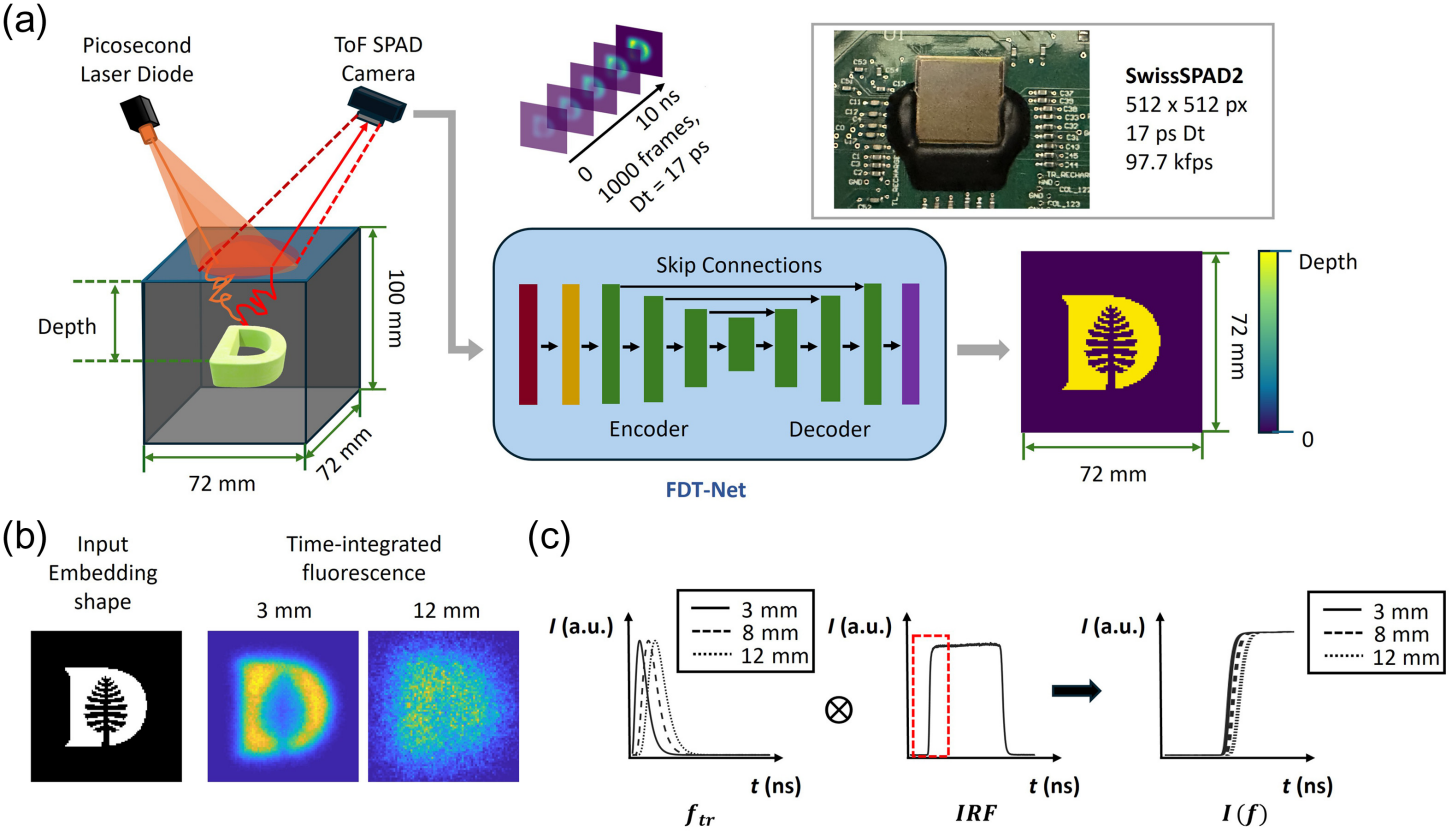
Overall summary of this study. (a) Depth map reconstruction pipeline. For the input time-resolved data, Dt is the time-gate step between two subsequent gate positions, fixed to 17.857 ps. The SwissSPAD2 sensor has 512×512  pixels, whereas in this work, we use half of the array (256×512  pixels). (b) Time-integrated fluorescence imaging at different inclusion depths. (c) Time-resolved fluorescence intensity profiles showing how inclusion depth affects signals. The fluorescence response is convolved with the IRF to get the real fluorescence intensity profiles.

In this work, we develop and demonstrate a fluorescence depth topography network (FDT-Net) optimized toward fast topology mapping. It is the first spatiotemporal model designed for the rapid conversion of time-resolved fluorescence image stacks to fluorescence depth maps utilizing flat-field illumination. Emphasis is put on optimizing the imaging scheme^33^ and reconstruction network to enable future translation into a real-time imaging application. The model is optimized for the SwissSPAD2 sensor images as input data. Training uses pre-validated optical Monte Carlo simulation datasets, incorporating the Monte Carlo eXtreme (MCX) environment and experimental IRF of our imaging setup (Fig. 1).^28^ We demonstrate the performance of FDT-Net with three systematic experiments: (1) fluorescence topology reconstruction in homogeneous media, (2) topology reconstruction with background fluorescence, and (3) comparative evaluation against pixel-wise CNN and 3D-CNN architectures under identical conditions.

## 2 Network Architecture, Training, and Evaluation Method

### 2.1 Network Structure

**Fig. 2:**
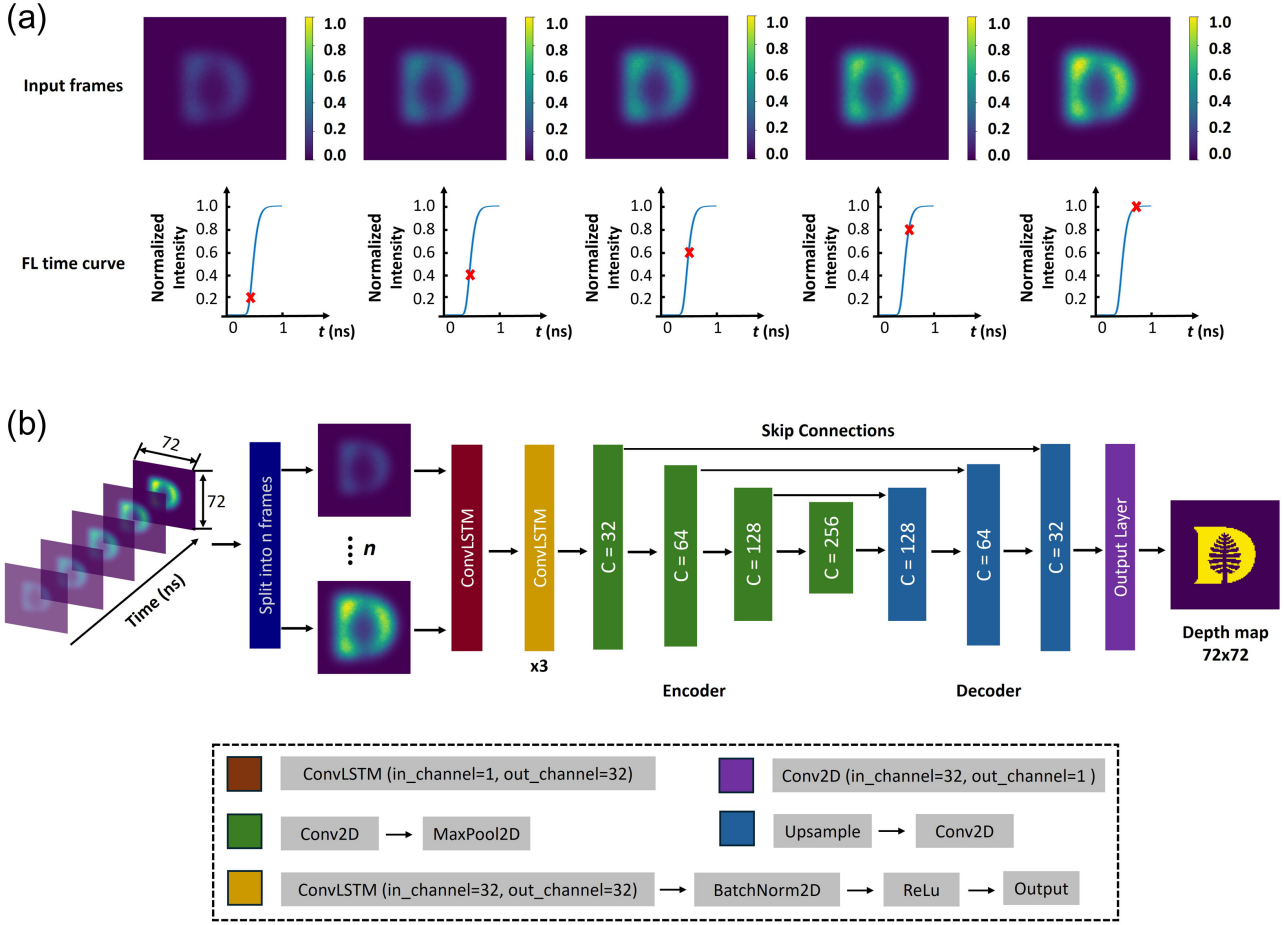
FDT-Net structure. (a) Input frames and corresponding time-resolved fluorescence curves. The responses are convolved with the IRF of the SwissSPAD2. (b) Architecture of the proposed spatiotemporal deep learning network (FDT-Net). The input data are an N×72×72×10 fluorescence video, where N is the total number of training datasets. Five example frames from the same input time-resolved fluorescence data are shown. C in each Conv2D blocks represents the output channels. The network output is the spatially resolved inclusion’s depth with the same size as the video frame.

The FDT-Net is designed to generate depth maps from time-resolved epi-illumination fluorescence image stacks recorded from the surface of optically diffusive and absorptive volumes. The topological structure of FDT-Net is divided into two primary components: first, a convolutional long-short-term memory (ConvLSTM) branch^34^ tasked with the extraction of spatial and temporal features, and second, a U-Net-like structure^35^ that functions as the generator, producing the depth map. Figure 2 shows the general architecture of the FDT-Net.

In contrast to prior works^30^^,^^36^ that focused on extracting either pure temporal or spatial information, this network was designed to capture both spatial and time-resolved information from simulated fluorescence datasets. For the network design, we used a ConvLSTM network to process fluorescence time-sequence datasets. ConvLSTM was chosen over Conv3D architectures for its ability to maintain hidden states across time steps, enabling efficient capture of long-range temporal dependencies in fluorescence dynamics.^37^ This recurrent design is particularly suited for modeling sequential fluorescence processes and capturing temporal correlations in intensity profiles. CNNs are well known for their efficacy in pattern and image learning tasks. In the first ConvLSTM block, we employed a kernel size of 5 to capture spatial features across the entire image at each time point. A larger kernel size was expected to learn more global information such as spatial connections. Subsequent ConvLSTM blocks used a reduced kernel size of 3 to extract more detailed features. The ConvLSTM model used convolutional operations instead of matrix multiplications in recurrent layers to learn temporal dynamics while preserving the spatial arrangements of the original video content.

Three sequential ConvLSTM layers were integrated into the network to capture the critical temporal changes of fluorescence intensity. The ConvLSTM output was then fed into U-Net layers, functioning as a generator. U-Net was chosen for its ability to discern both global and local features within both whole time-resolved fluorescence datasets and individual frames. This structure enhanced the precision of predicted details, particularly along the edges, and ensured that the dimensionality of the reconstructed depth map matched that of the input video frames.

The loss function we used here was a weighted combination of L1 and L2 losses, defined as:

$$L_{\mathrm{FDT}-\text{Net}}=L_{1}+\alpha L_{2},$$

where $L_{1}$ represents the mean absolute error loss, $L_{2}$ represents the mean square error loss, and α is the weight factor for $L_{2}$ loss. The $L_{1}$ loss aimed at preserving detailed information within the prediction results, with a specific emphasis on sharply delineating pattern edges. Conversely, the $L_{2}$ component of the loss function served to impart a smoothing effect on the predictions by averaging squared deviations, thereby facilitating noise reduction in the generated depth maps. The choice of the weight factor α was guided by our intention to prioritize the achievement of clear-edge delineation in the predicted outputs. During the training steps, α was set to 0.5, which provides the best overall results. A comprehensive evaluation of different α values’ influences on reconstruction results is included in Fig. 10.

### 2.2 Network Training Setup

To ensure the robust performance of our network, 5000 different wide-field time-resolved fluorescence intensity data were generated using the MCX toolbox in MATLAB^38^ for model training. Training and validation data were split in a 9:1 ratio. The simulation volume size was chosen as $72\times 72\times 100\;{\mathrm{mm}}^{3}$ to model the wide field-of-view typical in clinical FGS and match our experimental setup. One or several fluorescence inclusions (7:3 ratio) were included in the geometry, featuring shapes derived from either EMNIST letters^39^ or from simple geometric forms such as squares or circles. The size of the inclusions varied between $16\times 16\;{\mathrm{mm}}^{2}$ and $32\times 32\;{\mathrm{mm}}^{2}$, and the inclusion thickness is set to 10 mm. The excitation and emission wavelengths were set to 635 and 680 nm, respectively, to mimic the IRDye680 fluorophore. The fluorescence lifetime was set to 0.1 ns. The Jacobian function was calculated at the surface for each volume to obtain the 3D time-resolved fluorescence signals *in silico*.^29^

The simulation used a constant medium absorption coefficient of $0.02\;{\mathrm{mm}}^{-1}$ and a scattering coefficient of $4.77\;{\mathrm{mm}}^{-1}$, which corresponds to 1% intralipid-PBS suspension (g=0.73 and n=1.37) used in the experimental validation. The inclusion depths and background fluorophore concentrations were changed in the simulation to emulate environments with homogeneous tissue optical properties and nonzero background fluorescence.^2^ The depth of the inclusion varied from 1 to 15 mm, and the background fluorophore concentrations varied between 0% and 10% of the fluorophore concentration in the inclusion. For each of the 5000 different simulation cases, the MCX model generated time-resolved 3D fluorescence fluence stacks with 300 time gates at a temporal step size of 17 ps. The step size was chosen according to the time delay step size of the SwissSPAD2 camera gating. Finally, the fluence map on the uppermost layer (surface) of each 3D fluence map represented the image as captured by the camera. These 2D fluence maps were arranged into an (x,y,t) data cube.

To optimize GPU memory usage, we developed a method for down-sampling fluorescence video data while preserving rising-edge features that are essential for depth recovery. Temporal fluorescence intensity profiles were first normalized per pixel to their maximum intensity across all time steps. Second, the temporal profiles of fluorescence were down-sampled to only 10 rising-edge indices; the time frame indices of fluorescence rising edges were recorded per pixel at intensity thresholds of 0.1 to 1.0 with a step size of 0.1. These 10 index frames formed a new array, which represented the final training input data. The normalization step was implemented to accelerate the training process and ensure consistency across the training dataset; original integral intensity values were kept for possible future use in a concentration calibration step.

The model used rectified linear units (ReLU) as the activation function and the Adam optimizer with a learning rate of 3e–5 for optimization. Batch normalization was applied after each ConvLSTM layer to stabilize and accelerate the training process. The model was trained for a maximum of 240 epochs with a batch size of 32, and early stopping was applied based on validation loss to prevent overfitting, with training typically converging well before the maximum epoch limit, as shown in Fig. 11. The deep learning model was implemented within the PyTorch framework, and all experiments were carried out on a Windows system workstation equipped with an NVIDIA RTX 2080-Ti GPU and 12 GB RAM. Full training hyperparameters are summarized in Table 1.

### 2.3 Experimental Setup and Network Training Data Validation

**Fig. 3:**
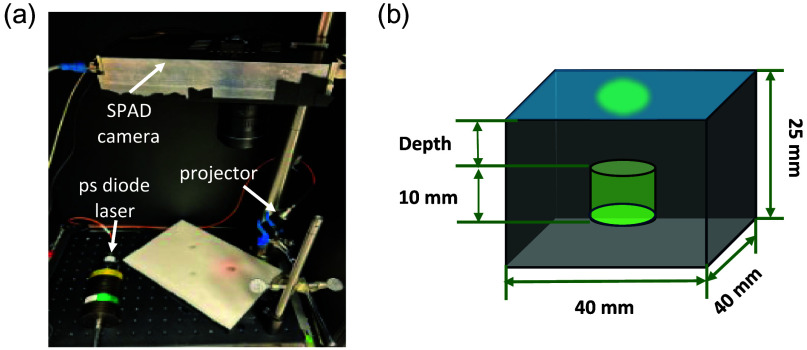
Experimental and simulation configurations. (a) Laboratory setup showing the SPAD camera system, picosecond diode laser source, and projector for fluorescence measurements. (b) Schematic of the Monte Carlo eXtreme (MCX) simulation geometry, illustrating the fluorophore inclusion depth and thickness parameters.

To validate the accuracy of our simulated training datasets as compared to the real fluorescence intensity response, we simulated and imaged a range of optical tissue phantoms with known optical properties. The experimental setup is shown in Fig. 3(a). The system consisted of a 2D single-photon avalanche diode (SPAD) array camera^40^ (SwissSPAD2, EPFL, Switzerland) synchronized with a 635 nm, 20 MHz picosecond excitation laser (LDH-D-C-635 M diode and PDL 828 driver, Picoquant, Germany). The SPAD camera was synchronized to the laser using the main sync output of the laser controller and fast comparator (MAX961, Maxim Integrated) as a level translator. This signal was converted to a+5  V TTL output and provided as input to the camera’s field-programmable gate array (Opal Kelly XEM7360, Xilinx, California, United States). The phantom consisted of a plastic slab of calibrated optical properties (INO, Quebec, Canada; $\mu_{a}=0.0141\;{\mathrm{mm}}^{-1}$, $\mu_{s}^{\prime }=1.2004\;{\mathrm{mm}}^{-1}$) with machined holes as reservoirs for liquid fluorophore solution. We used IRdye680 RD fluorophore (Li-COR Bioscience, Lincoln, Nebraska, United States) at a concentration of 10  μmol/L, mixed with 2.5% intralipid as a scattering component. The slab was covered with a plastic slab of the same material and a thickness of 1 to 8 mm. For the purpose of MCX validation, the simulation parameters and geometry were configured to match the dimensions and optical properties of the INO phantom shown in Fig. 3(b).

### 2.4 Test Dataset and Evaluation Metrics

To evaluate model performance, two distinct test sets were designed, each targeting a different experimental condition. For the homogeneous media experiment (Sec. 3.2), 15 newly generated fluorescence geometries were used as test cases, featuring one or two inclusions, including letters from the EMNIST dataset and simple geometric patterns. Critically, none of these geometries appeared in the training or validation datasets, ensuring an unbiased evaluation of model generalizability across different inclusion shapes. Each of the 15 geometries was evaluated across five depths ranging from 5 to 15 mm, yielding a total of 75 test datasets. Reconstruction accuracy was quantified using the mean absolute error (MAE) computed pixel-by-pixel between the predicted and ground truth depth maps. To isolate performance at clinically relevant locations, inclusion-specific metrics were additionally computed exclusively over pixels within the ground truth binary mask: inclusion-region MAE, root mean square error (RMSE), and mean error (ME, i.e., signed bias). The Dice score is used to assess the spatial shape preservation of the reconstructed inclusion boundaries.

For the background fluorescence experiment (Sec. 3.3), multiple test datasets were generated with varying inclusion shapes and background-to-inclusion fluorophore concentration ratios ranging from 5% to 20% of the inclusion concentration, covering the most common clinical scenarios. Inclusion depths were set at 3 mm to simulate surface-level cases. Model performance was quantified using Dice score and structural similarity index (SSIM) metrics to assess both shape preservation and overall image quality of the reconstructed depth maps.

For the comparative evaluation against baseline architectures (Sec. 3.4), FDT-Net was benchmarked against two baseline models, representing widely used approaches in the field. The first baseline is a single pixel-wise CNN model, which processes only temporal information at each pixel independently while ignoring spatial correlations between neighboring pixels. The second baseline is a Conv3D model, which applies identical convolutional kernels uniformly across both spatial and temporal dimensions without explicitly modeling sequential temporal dependencies. All three models were evaluated on identical test datasets under both clean signal and background fluorescence conditions to ensure fair comparison.

## 3 Results and Discussion

### 3.1 Training Data Validation

**Fig. 4:**
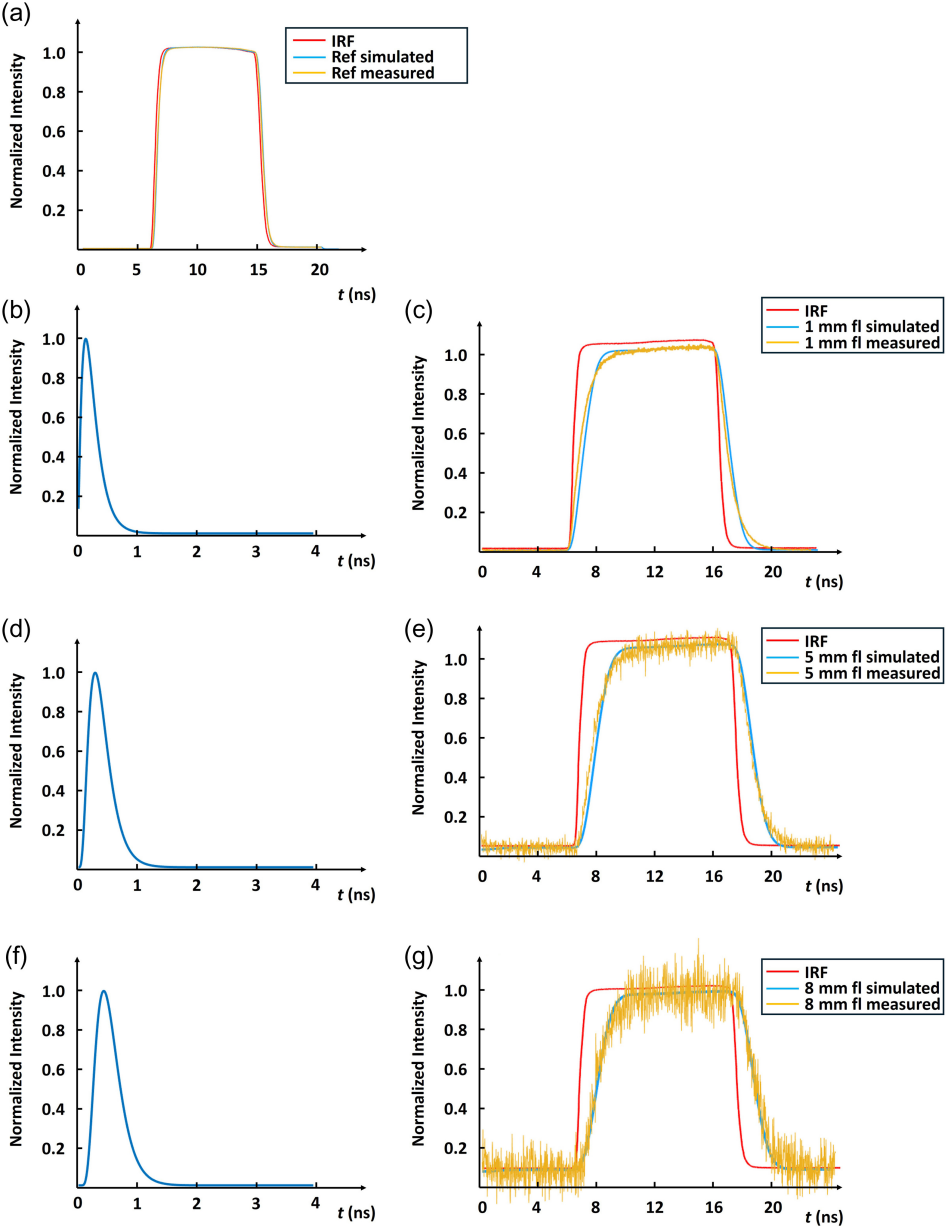
(a) Surface reflectance temporal intensity profile comparing MCX simulation result and real measurement. Panels (b), (d), and (f) show MCX-simulated time-resolved fluorescence responses at depths of 1, 5 mm, and 8 mm, respectively, without instrument response function (IRF) convolution. Panels (c), (e), and (g) compare experimental and MCX-simulated time-of-flight fluorescence intensity profiles measured at the surface, with fluorophores embedded at corresponding depths.

Figure 4 demonstrates the validation of the MCX simulation framework against experimental measurements. Figure 4(a) compares the simulated versus measured time-resolved reflectance intensities at the reference surface. Figures 4(b), 4(d), and 4(f) present the simulated responses for fluorescent inclusions at 1, 5, and 8 mm depths, respectively. The corresponding Figs. 4(c), 4(e), and 4(g) compare the MCX-simulated fluorescence responses with the actual SPAD camera measurements at these depths. The measured response overall matched the MCX simulation, confirming the general accuracy of the MCX training dataset generator. Quantitative analysis of the similarities is shown in Table 2. The MCX simulation yielded a slightly faster fluorescence response than the measured one, likely due to the fluence sampling from the uppermost layer of tissue rather than an external in-air detector, which was optimized for simulation efficiency. The 10 ns IRF shown in Fig. 4 is typical for the SwissSPAD2 class of detectors.^40^

### 3.2 Recovering Sub-Surface Topology of Distributed Fluorescence Inclusion in Nonfluorescent Media

**Fig. 5:**
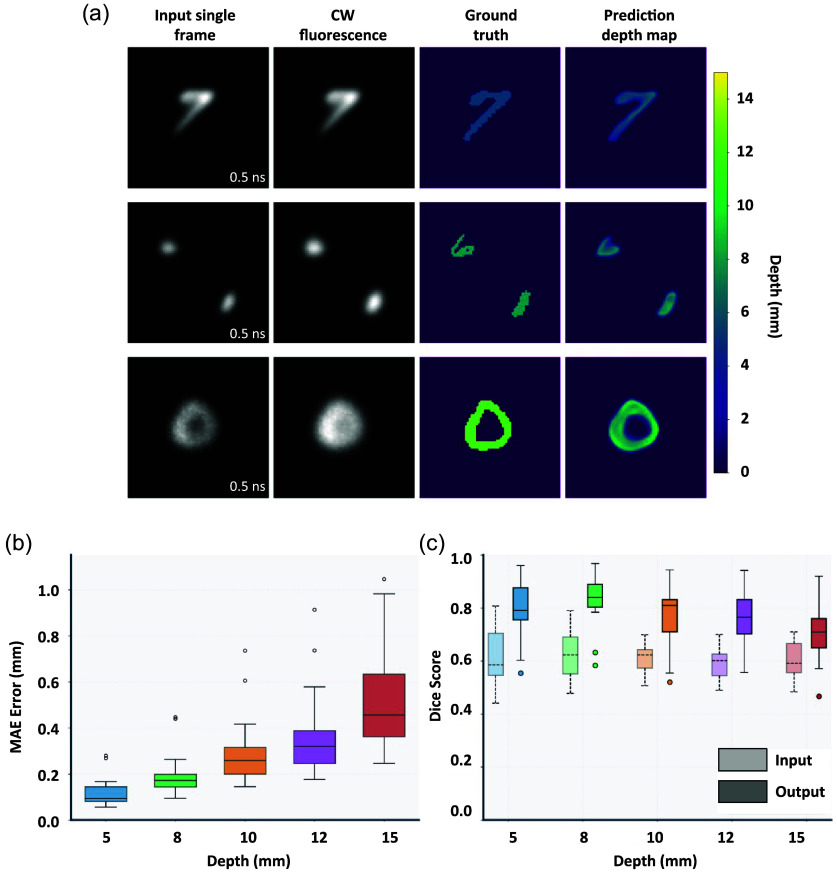
Model performance evaluation across varying depths. (a) Representative depth map predictions at three depths, showing single input time-resolved frames (0.5 ns), CW fluorescence, ground truth, and predicted depth maps. (b) Mean absolute error (MAE) analysis of depth predictions for 15 distinct patterns across depths ranging from 5 to 15 mm. (c) Comparison of Dice scores between input frame (light) and predicted depth map (dark) relative to ground truth.

This experiment evaluated the network’s ability to accurately recover the true topological structure of fluorescent inclusions at various depths beneath the tissue surface, where light scattering typically distorts their apparent geometry. As shown in Fig. 5(a), the inputs and predicted depth maps of three distinct geometry types are shown next to their ground truths. Visually, the shapes are in good agreement, exhibiting stable edge recognition compared with the original noisy and diffused input signals. Quantitatively, the Dice score between the ground truth and the reconstructed depth maps is in the range of 0.71 to 0.86, significantly higher than the scores of 0.58 to 0.67 between the original input and the ground truth.

As shown in Fig. 5(b), when the inclusion depth was less than 10 mm, the MAE remained below 0.4 mm. However, as the depth increased, so did the MAE. This occurs because the captured fluorescence signal intensity diminishes at the surface, and each input fluorescence video becomes increasingly blurred with deeper inclusion depths. Overall, the sub-millimeter MAE values validated the model’s accuracy and sensitivity in spatial prediction across varying geometries.

**Fig. 6:**
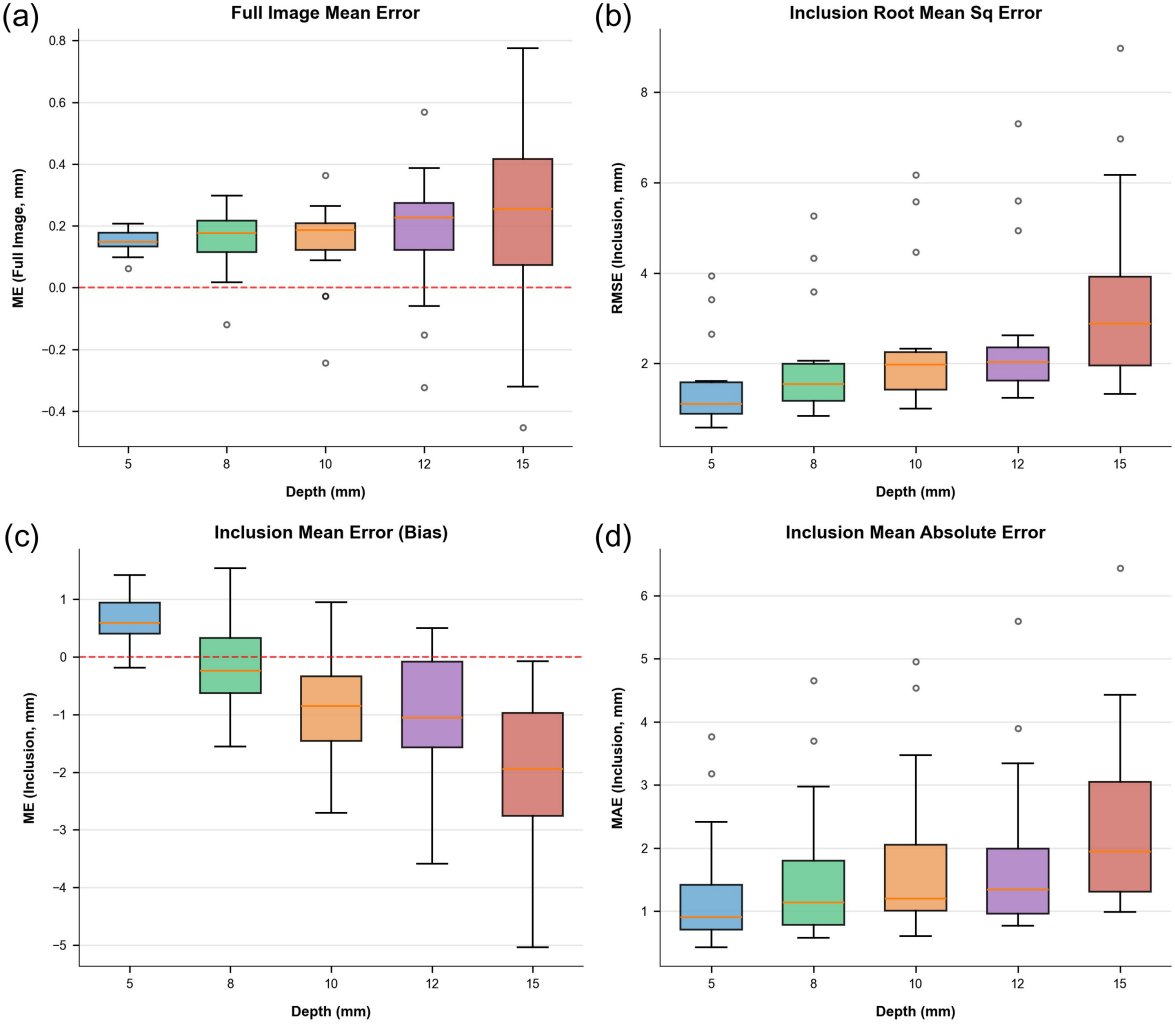
Model performance evaluation on inclusion focused region. (a) Full image ME computed over all pixels, showing consistently low bias across depths. (b) Inclusion-region RMSE, computed exclusively over pixels within the ground truth inclusion mask. (c) Inclusion-region ME. The red dashed line indicates zero bias. (d) Inclusion-region MAE.

To further characterize reconstruction accuracy at clinically relevant locations, we evaluated error metrics exclusively within the inclusion region defined by the ground truth binary mask. As shown in Fig. 6, the inclusion-region MAE is ∼0.8  mm at 5 mm depth and remains typically around 1 mm for inclusion depths up to 12 mm, and the RMSE follows a similar trend. The inclusion-region bias reveals that the network slightly overestimates depth for shallow inclusions (median ME≈+0.6  mm at 5 mm) and progressively underestimates depth at greater depths (median ME≈−1.0  mm at 12 mm). This systematic shift is consistent with the reduced fluorescence signal intensity reaching the surface from deeper inclusions, which limits the temporal contrast available for accurate depth recovery. Importantly, the inclusion-region MAE remains within the typical surgical margin range of 2 to 10 mm across all tested depths.

### 3.3 Recovering Sub-Surface Topology of Fluorescence Inclusion in Media with Background Fluorescence

**Fig. 7:**
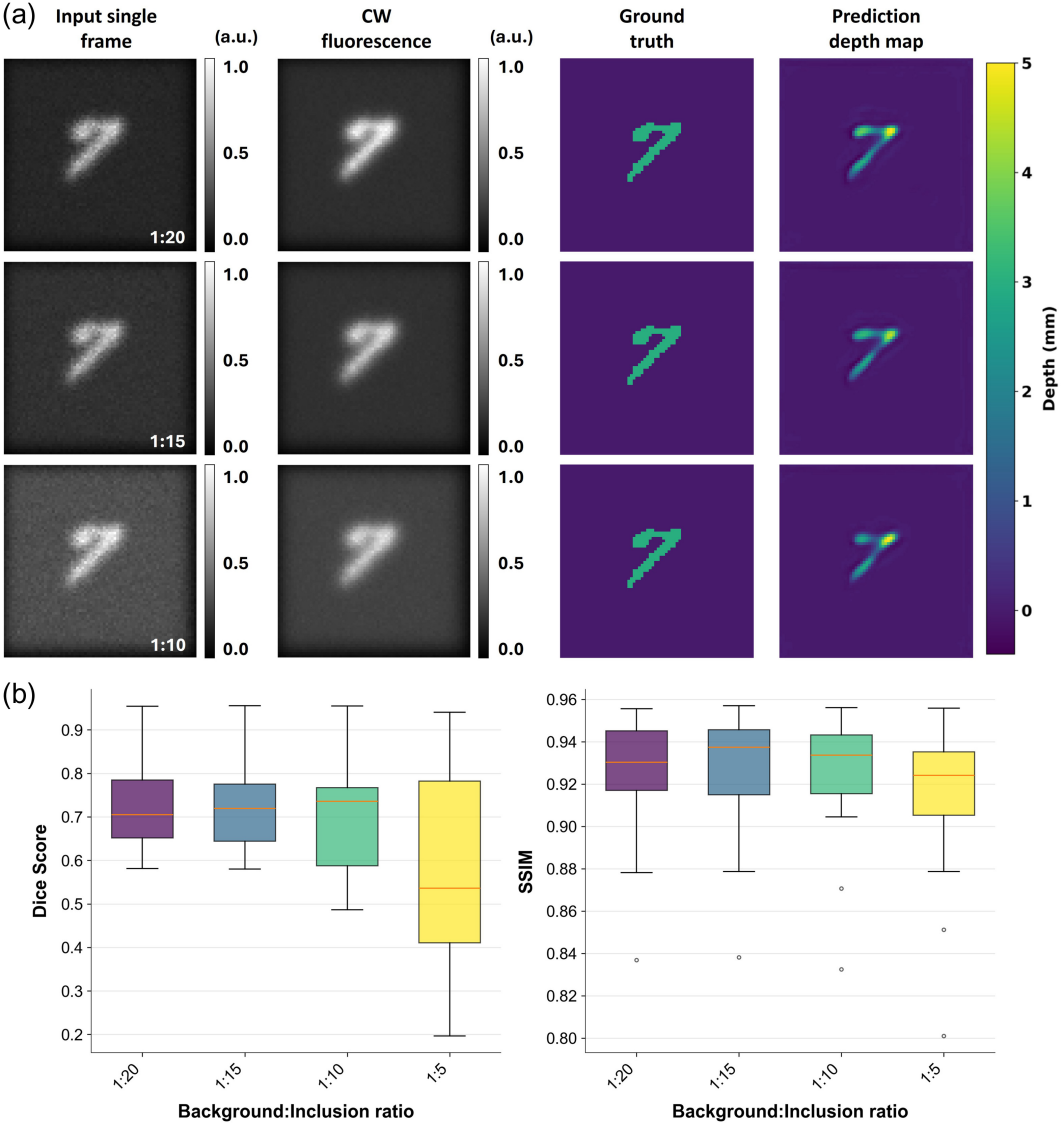
Model performance evaluation across varying background fluorescence concentrations. (a) Representative depth map predictions for a single test geometry at three background concentration levels (1:20, 1:15, and 1:10), displaying the input time-resolved frame, corresponding CW fluorescence image, ground truth depth map, and FDT-Net predicted depth map. (b) Dice score and SSIM distributions comparing predicted depth maps against ground truth across varying background fluorescence concentrations.

This experiment evaluated FDT-Net’s performance in recovering fluorescent inclusion topology within media containing background fluorescence. Figure 7(a) presents representative results for a single test geometry under three different background concentration levels. The predicted depth maps are displayed alongside their corresponding ground truths, demonstrating strong visual agreement in reconstructed shapes across all noise conditions. Figure 7(b) provides quantitative validation through Dice score and SSIM metrics across the tested background concentrations. FDT-Net achieves median Dice scores above 0.70 and median SSIM values consistently above 0.93 for background-to-inclusion ratios up to 1:10, demonstrating robust topology reconstruction capability in the presence of substantial background interference.

### 3.4 Comparative Evaluation Against Conventional Architectures

**Fig. 8:**
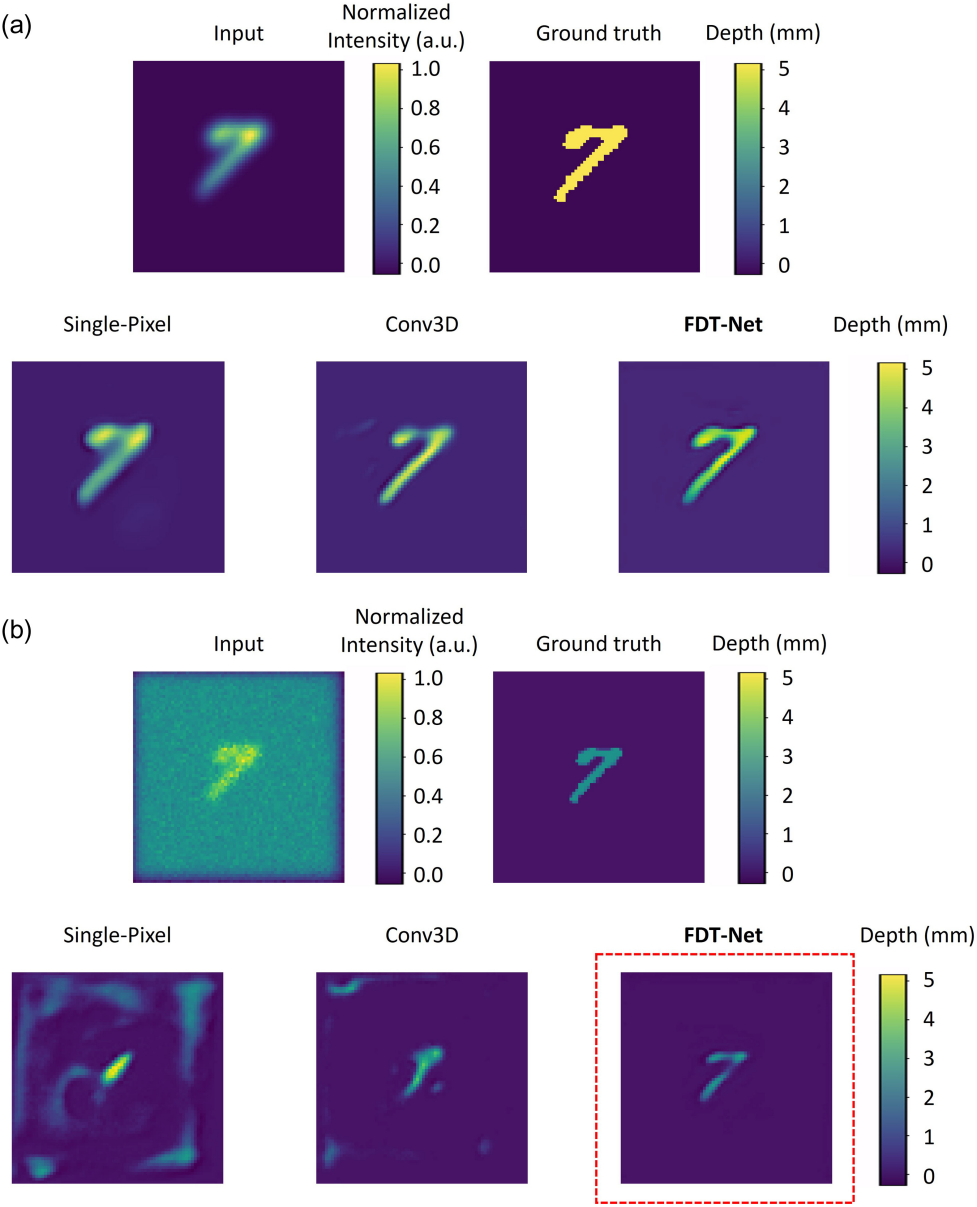
Reconstruction performance comparison under different signal conditions. (a) With clean input signals containing only fluorescence inclusion information, all three methods successfully recover the true topology, though Conv3D and FDT-Net produce sharper boundaries through joint spatiotemporal processing. (b) In the presence of background signals (background: inclusion = 1:10), only FDT-Net accurately reconstructs the inclusion topology and spatial distribution, whereas Single-Pixel and Conv3D methods fail to distinguish the true fluorescence signal from background interference.

Figure 8 illustrates a comparative performance of FDT-Net against the two baseline architectures described in Sec. 2.4, evaluated under different background signal conditions. When no background signal is present and photons originate solely from fluorescent inclusions, all three models successfully recover the topology. Notably, the two models incorporating spatial information (FDT-Net and Conv3D) produce reconstructed edges with reduced blurriness compared with the single pixel-wise model, highlighting the critical importance of spatial information in achieving high-fidelity reconstructions. However, as shown in Fig. 8(b), when background signals are introduced, only FDT-Net maintains accurate topology reconstruction capability.

### 3.5 Discussion

The proposed FDT-Net trained with Monte Carlo simulations demonstrated robust performance, as shown in the different test scenarios. A key architectural choice was using ConvLSTM rather than Conv3D for temporal feature extraction. Unlike Conv3D, which treats all temporal frames uniformly, ConvLSTM explicitly models sequential dependencies through gating mechanisms, allowing the network to capture the progressive temporal dynamics of photon propagation. Moreover, ConvLSTM processes temporal sequences recurrently with fixed parameters, avoiding the need for large 3D kernels that would be required to capture long-range temporal dependencies, which leads to faster training and better generalization.

The network achieves a 25 ms topology reconstruction time per input on the Nvidia RTX 2080Ti platform (Table 3). Such performance enables integration into the existing image processing pipeline in an on-site *exvivo* tumor margin assessment. With moderate upgrades of GPUs and computational optimization, the architecture could even support real-time applications. The acquisition time of real-time imaging now is around 10  μs per frame, but this requires an optimized acquisition scheme with a limited number of acquired temporal gates to maintain a reasonable framerate. We demonstrated the performance of our network, which involved only 10 temporal gates as data input. This threshold-based sampling captures when each pixel reaches successive intensity levels from initial response to peak, preserving temporal information about spatial heterogeneity in response timing. Such an approach provides a robust representation that enables the ConvLSTM model to extract time-dependent features from the temporal dynamics.

Using such inputs, the model performed with an accuracy of less than 0.2 mm at depths of 5 mm, increasing to less than 0.8 mm at depths of 12 mm. These accuracies are better than traditional fitting methods and also match the typical minimum margin range of 2 mm to 1 cm in tumor resections.^41^^–^^43^ Submillimeter depth accuracy is clinically significant because tumor margins are often within 1 to 2 mm of critical structures or healthy tissue. Overestimating depth by even a few millimeters could lead to unnecessary removal of functional tissue, particularly in neurosurgery, where excessive resection may cause neurological deficits, or in breast-conserving surgery, where it affects cosmetic outcomes.^44^^,^^45^ Conversely, underestimating depth risks leaving residual tumor cells that increase recurrence rates. Current surgical tools, including robotic systems and intraoperative navigation platforms, can integrate depth information through overlay displays.^46^^,^^47^ This might allow the potential usage of millimeter-level depth maps for guiding tumor resection in real time. Balancing the weight factor in the loss function also provided a tunable edge enhancement functionality, which worked in our case of sharply defined fluorescence inclusion boundaries but may have to be adjusted in more general cases, which include diffuse fluorescence margins.

The model still includes several obvious simplifications and exhibits its limits. First, we used prior knowledge of tissue optical properties and assumed homogeneous distributions. Real-world scenarios typically include tumor and nontumor regions, which usually exhibit heterogeneous optical properties. To address this, the training datasets need to be expanded in several dimensions. First is to broaden the range of optical properties to include $\mu_{a}$ from 0.001 to $0.2\;{\mathrm{mm}}^{-1}$ and $\mu_{s}$ from 1.5 to $20\;{\mathrm{mm}}^{-1}$, representing typical human tissue values.^48^^,^^49^ The training will incorporate multiple fluorophores with diverse temporal characteristics, including fast-response dyes such as ICG, IRDye680, and fluorescein. Importantly, the training geometries will be modified to include heterogeneous optical properties, where background tissue and inclusions exhibit different optical characteristics. Moreover, to extend our model to potential clinical applications, additional measurements of these heterogeneous optical properties or their surrogates will be necessary. Incorporating time-resolved reflectance could provide the model with this extra information,^50^ leading to more accurate reconstruction results and improved generalizability.

Beyond optical property assumptions, both the air-tissue boundary and sub-surface inclusion surfaces were assumed to be flat and planar, whereas real surgical scenarios typically involve irregular tissue surfaces and complex tumor geometries. Expanding the simulation training datasets to include curved surface geometries and potentially fine-tuning the model with real experimental data represent important future steps toward clinical translation.

**Fig. 9:**
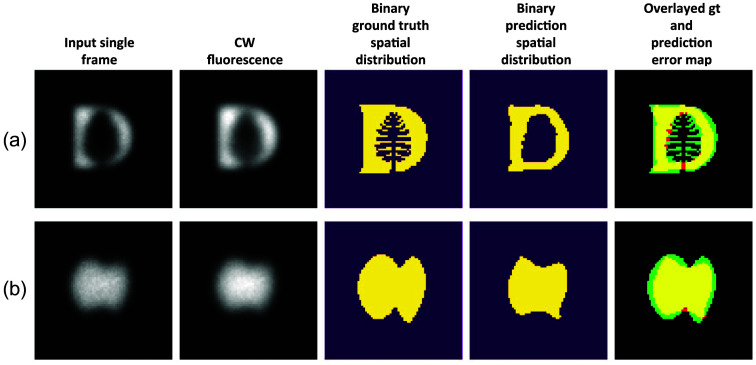
Additional test cases for spatial distribution prediction. (a) Complex inclusion with tree-like pattern at 5 mm depth (32×32  pixels), showing input frame, CW fluorescence, binary ground truth, model prediction, and error map overlay. (b) Brain tumor–shaped inclusion at 10 mm depth (32×32  pixels), demonstrating partial loss of fine spatial features in the prediction compared with ground truth.

We also observed that the input data reached physical limits for certain spatial features due to the spatial resolution of the camera, where the network tends to lose certain levels of spatial detail as shown, e.g., in Fig. 9. This performance limit could be addressed to a certain degree with a ballistic photon approach.^51^^,^^52^ However, this approach will likely be limited by the 0.5 to 4 ns decay times of pre-clinical or clinically usable fluorophores.^53^^–^^55^ In addition, increasing the temporal resolution would lead to a worse signal-to-noise ratio per frame, which may already be quite limited by the current sensor technology.^40^

## 4 Conclusion

In this study, we proposed a spatiotemporal deep learning network to recover the depth and shape of fluorescent inclusions embedded in tissue as dense optically diffusive media, for the purpose of sub-surface depth estimation during surgical image guidance. A large number of MCX-simulated time-resolved fluorescence sequences were used to train the model, which was subsequently tested on datasets featuring varying inclusion shapes and depths. The model resolved depth information from 2 mm with a MAE of less than 0.2 mm to 12 mm with a MAE of less than 0.8 mm. Although we limited the training cases to sharply defined fluorescent inclusions and homogeneous optical properties, which restricted the model’s applicability, we demonstrated the ability of the DL model to recover sub-surface nodules based only on spatiotemporal fluorescence image stacks. Future steps will incorporate time-resolved diffuse reflectance data as input and expand training datasets with optically heterogeneous inclusion media. The proposed model demonstrated its potential to rapidly resolve quantitative depth information, replacing analytical reconstruction models toward faster and more accurate image guidance in fluorescence-guided surgery.

## 5 Appendix

### 5.1 FDT-Net Performances with the Change of L2 Loss Weight

**Fig. 10:**
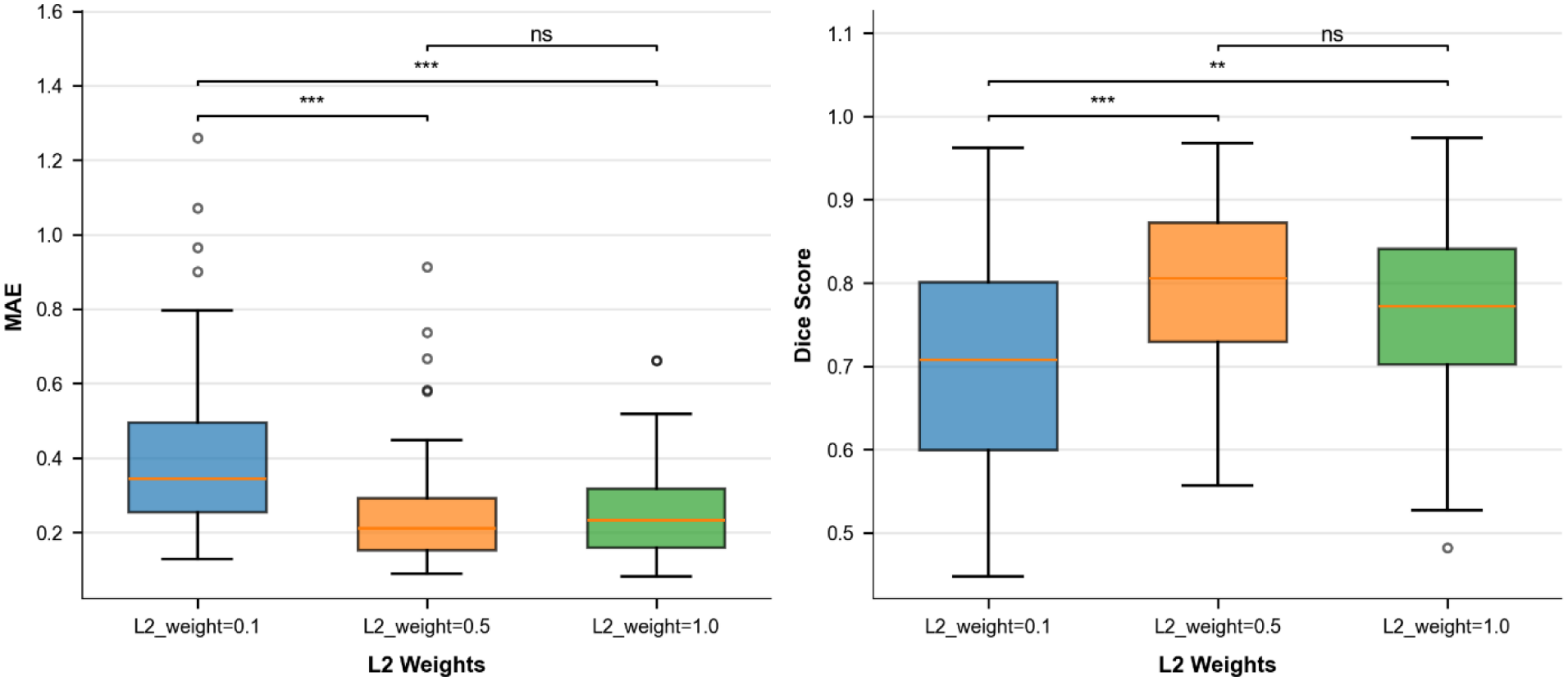
FDT-net performances with the change of L2 loss weights.

Figure 10 presents a quantitative comparison of model performance across varying L2 loss weights. Based on these results, an L2 weight of 0.5 is selected during training process.

### 5.2 Model Training and Validation Loss

**Fig. 11:**
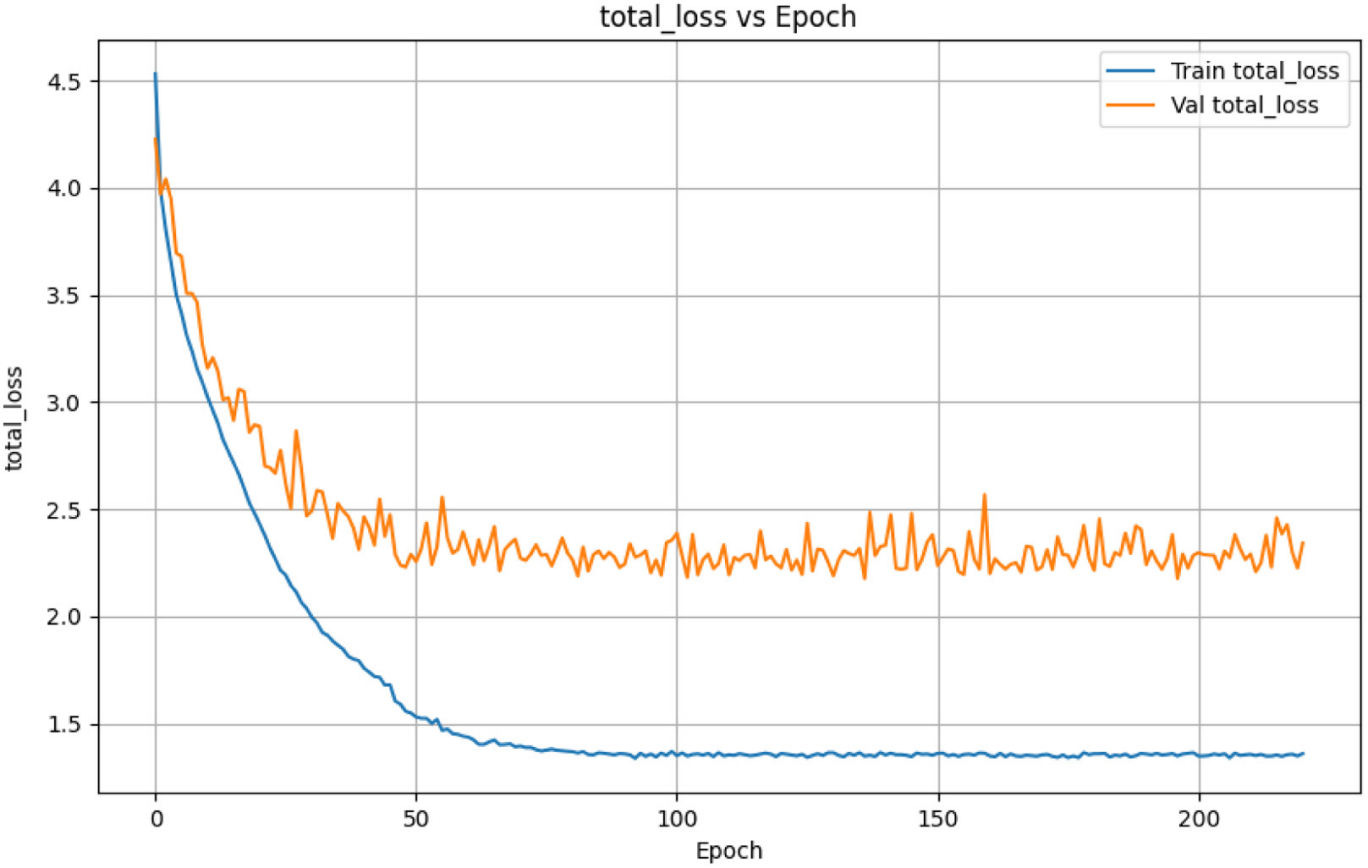
FDT-net training and validation loss curves.

Table 1 shows the parameters used in the model training process. Figure 11 shows the training and validation loss of FDT-Net. The model is converged after around 80 epochs.

**Table 1:** Model training parameters.

Number of data	5050
Batch size	32
Number of epochs	240
Activation function	ReLU
Optimizer	Adam
Learning rate	3e–5
Scheduler	ReduceLROnPlateau
Weight decay	1e–6
Minimum learning rate	1e–8

### 5.3 FDT-Net and Traditional Method Performances on Depth Reconstruction

**Fig. 12:**
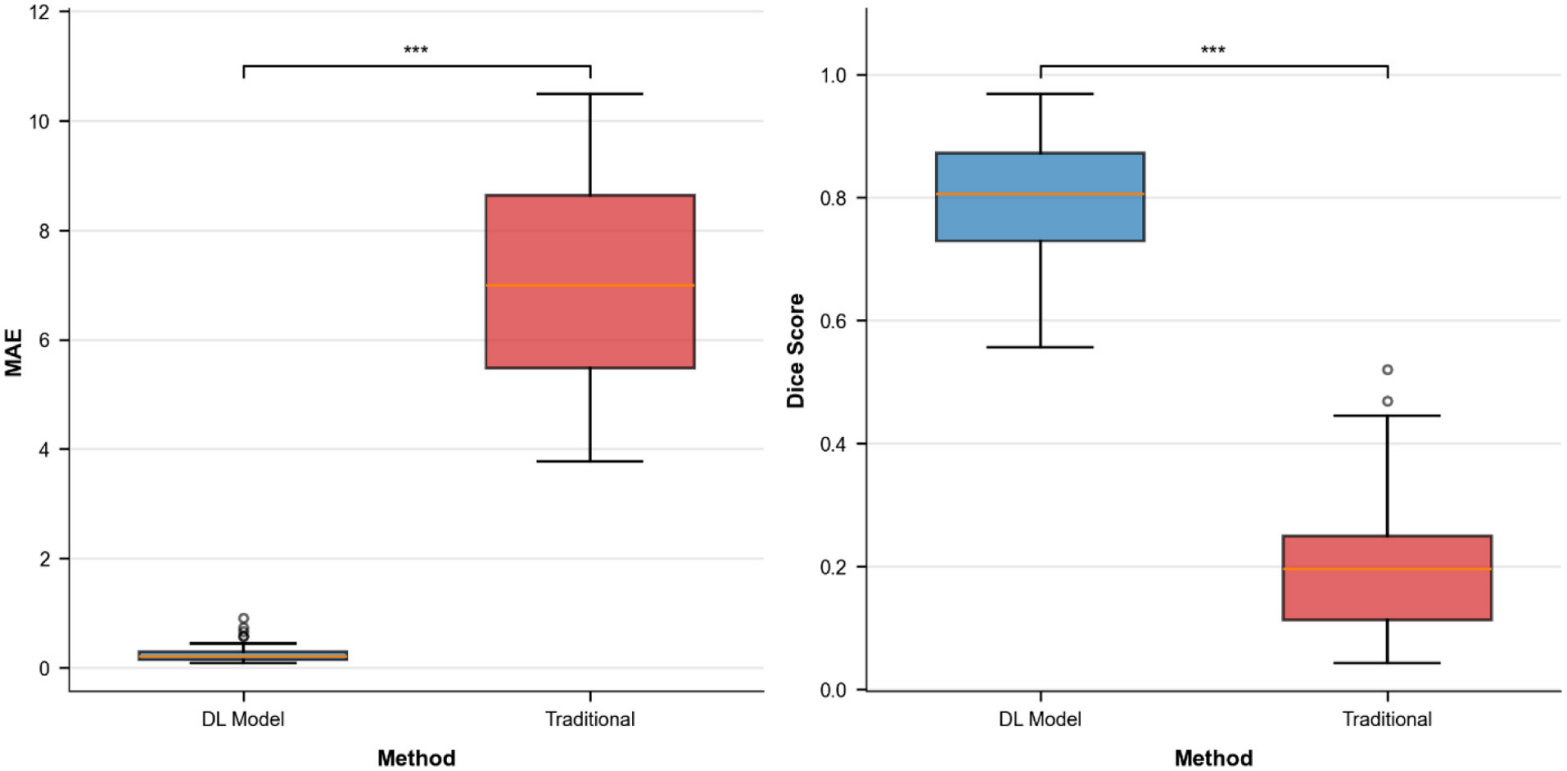
FDT-net and traditional method performances on depth reconstruction.

The key idea of the traditional method is that deeper fluorophores have longer photon travel times. Physics-based models usually have lower resolutions and worse boundary delineations (Fig. 12).

### 5.4 Deep Learning Model Performance Comparisons

**Fig. 13:**
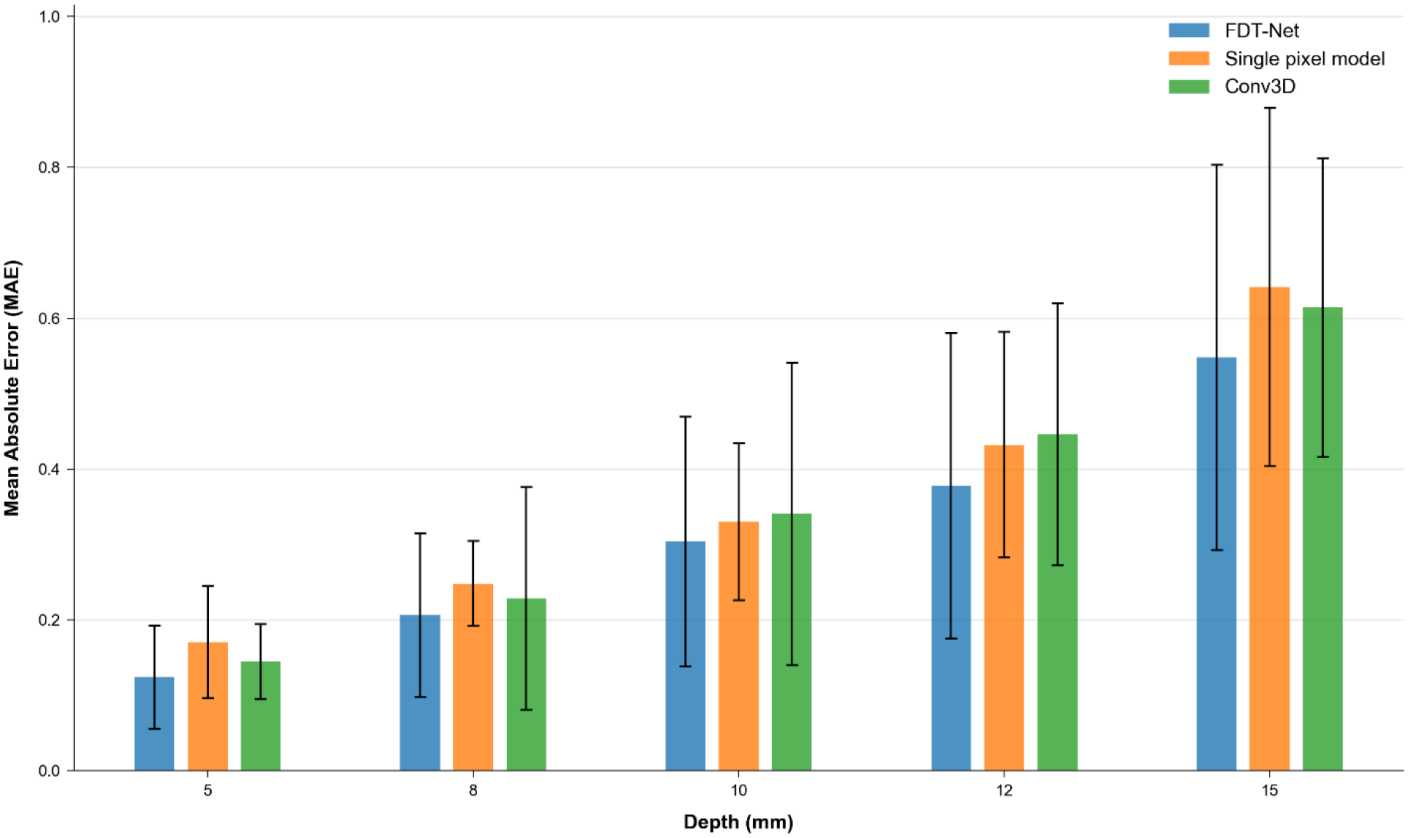
Model performance comparisons on different depths including single-pixel model, Conv3D model, and FDT-net.

Figure 13 shows a quantitative comparison of three architectures: the single-pixel model, Conv3D, and the proposed FDT-Net. FDT-Net consistently achieves better performance across all evaluated depths.

### 5.5 MCX-Simulated Time-Resolved Fluorescence Responses

**Fig. 14:**
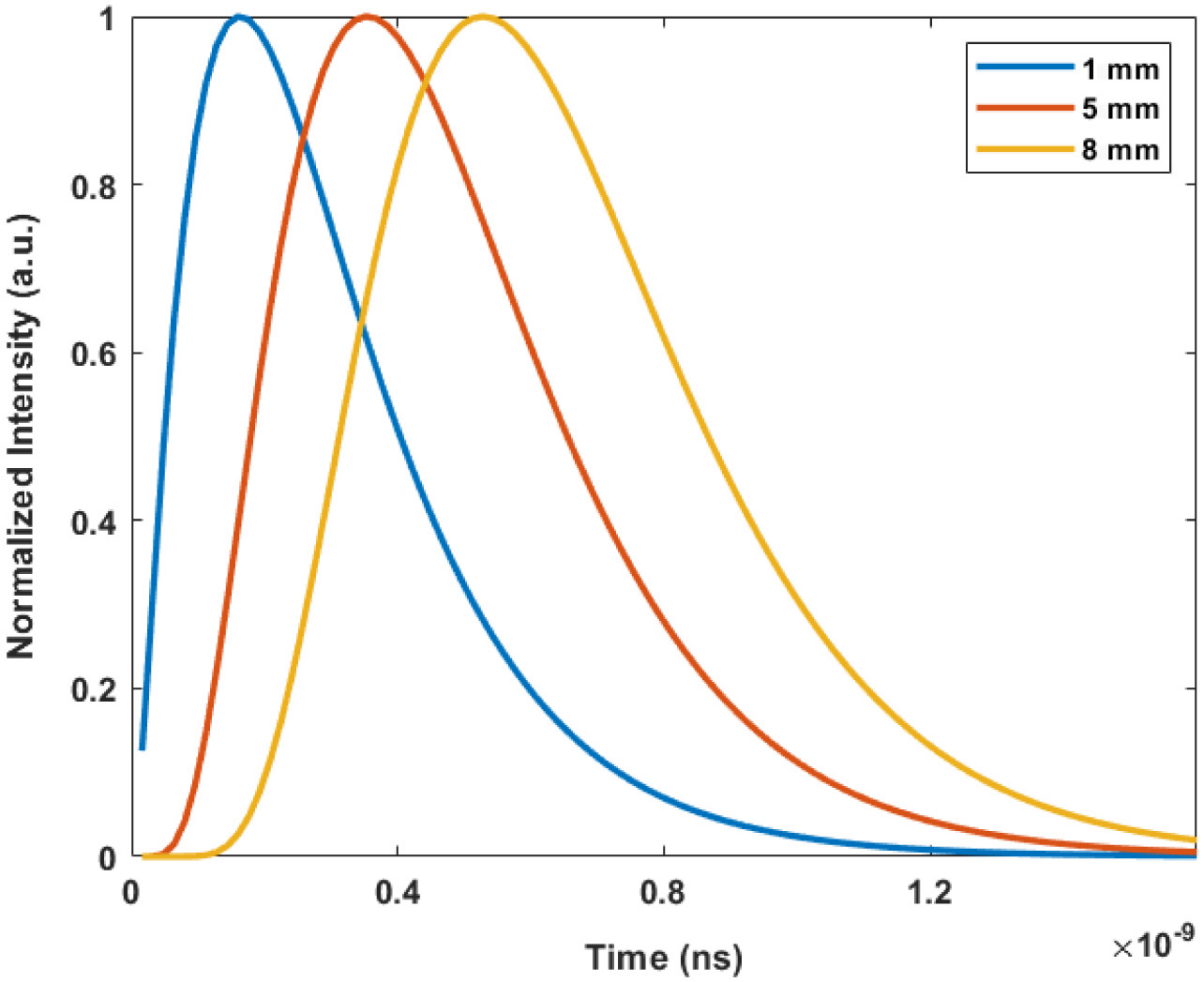
MCX-simulated time-resolved fluorescence responses at depths of 1, 5, and 8 mm.

Using an absorption coefficient of $0.02\;{\mathrm{mm}}^{-1}$ and a scattering coefficient of $4.77\;{\mathrm{mm}}^{-1}$, which correspond to 1% intralipid (g=0.73 and n=1.37), the peak shift from 1 to 8 mm is around 0.5 ns (Fig. 14).

### 5.6 Quantitative Analysis of Simulated/Experimental Time Profile Similarities

Three equations of similarity calculations are listed below:

1) Correlation: measures curve shape similarity ∈[−1,1].
  $$\mathrm{Corr}=\frac{\sum [(x_{i}-\overset{¯}{x})(y_{i}-\overset{¯}{y})]}{\sqrt{\left[\sum {\left(x_{i}-\overset{¯}{x}\right)}^{2}\times \sum {\left(y_{i}-\overset{¯}{y}\right)}^{2}\right]}},$$
  where $x_{i}$ is the measured points, $y_{i}$ is the simulated points, $\overset{¯}{x}$ is the mean of the measured data, and $\bar{y}$ is the mean of the simulated data.
2) $R^{2}$: measures how well the simulation explains real data variances ∈[0,1].
  $$R^{2}=1-\frac{\sum {\left(y_{i}-{\hat{y}}_{i}\right)}^{2}}{\sum {\left(y_{i}-{\bar{y}}_{i}\right)}^{2}},$$
  where $y_{i}$ is the measured points, ${\hat{y}}_{i}$ is the simulated points, and $\bar{y}$ is the mean of the measured data.
3) NRMSE: percentage error between curves.
  NRMSE=(∑(yi−y^i)2/nmax(yi)−min(yi))×100,
  where $y_{i}$ is the measured data points, ${\hat{y}}_{i}$ is the simulated data points, and n is the number of data points.

All three evaluation methods show that measured and simulated data are in good agreement. The variances are mainly from the noise of real measurements (Table 2).

**Table 2:** Quantitative analysis of simulated/experimental time profile similarities.

Inclusion depth (mm)	Correlations	$R^{2}$	NRMSE
1	0.974	0.862	0.1132
5	0.945	0.879	0.125
8	0.920	0.831	0.136

### 5.7 Model Inference Time Calculation

Below are the printed results in the terminal, the inference time is around 25 ms (Table 3).

**Table 3:** Model inference time calculation.

Model inference time code:
import time
start_time = time.perf_counter()
result = model.predict(input_data)
inference_time = time.perf_counter()-start_time
print(f“Inference time:{inference_time:.4f}seconds”)

**Table:** 

Inference time statistics
Total inferences measured: 600
Mean inference time: 0.0254±0.0033 s
Min inference time: 0.0206 s
Max inference time: 0.0306 s
Median inference time: 0.0274 s
Device: cuda
GPU: NVIDIA GeForce RTX 2080 Ti

## Data Availability

All data and Python routines are available upon request.
